# The Effects of a Sport-Based Training Program on Reaction Time and Fine Motor Coordination in Children with Autism Spectrum Disorder: A Pilot Study

**DOI:** 10.3390/sports14020080

**Published:** 2026-02-11

**Authors:** Fabiana Laurenti, Valentina Presta, Michela Compiani, Gianni Zobbi, Barbara Ilari, Maria Pia Picchi, Eugenia Maré, Federica Severini, Alessandro Guarnieri, Salvatore Mazzei, Orsola di Martino, Giulia Pozzi, Giancarlo Condello, Giuliana Gobbi

**Affiliations:** 1 Department of Medicine and Surgery, University of Parma, 43126 Parma, Italy; fabiana.laurenti@unipr.it (F.L.); valentina.presta@unipr.it (V.P.); alessandro.guarnieri@unipr.it (A.G.); salvatore.mazzei@unipr.it (S.M.); orsola.dimartino@unipr.it (O.d.M.); giulia.pozzi@unipr.it (G.P.); giuliana.gobbi@unipr.it (G.G.); 2 Department of Neuroscience, Biomedicine and Movement, University of Verona, 37124 Verona, Italy; 3 Unit of Sports Medicine and Cardiovascular Prevention, AUSL Reggio Emilia, Via Brigata Reggio, 24/1, 42124 Reggio Emilia, Italy; michela.compiani@ausl.re.it (M.C.); gianni.zobbi@ausl.re.it (G.Z.); barbara.ilari@ausl.re.it (B.I.); mariapia.picchi@ausl.re.it (M.P.P.); 4 Territorial Animation and Planning Area, Volunteer Service Center CSV Emilia, Viale Trento Trieste 11, 42124 Reggio Emilia, Italy; eugenia.mare@csvemilia.it (E.M.); federica.severini@csvemilia.it (F.S.)

**Keywords:** intellectual disabilities, neurodevelopmental disorders, physical activity

## Abstract

Background: Children with autism spectrum disorders (ASD) are generally less involved in physical activity and sport. Therefore, the present pilot study aimed at determining the effect of a sport-based training program on motor coordination development and functioning in children with ASD. Methods: Twenty children with ASD (age: 8.7 ± 1.6 years, 5 females) were included in a sport-based training program for 6 months. Participants were free to select their own sport discipline. Before and after the program, reaction time was evaluated using a simple (by identifying the targeted stimulus) and a complex (by discriminating the targeted stimulus among confounding signals) reactive test, while fine and gross motor coordination was assessed by transferring pennies, jumping in place (same sides synchronized), tapping feet and fingers (same side synchronized), and the Flamingo test. Results: The analysis showed a significant reduction (*p* = 0.016, d = 0.16) in complex reactive test (pre: 15.8 ± 14.8 s; post: 13.6 ± 11.1 s) and a significant improvement in transferring pennies test (pre: 6.3 ± 3.4 pt.; post: 7.8 ± 3.8 pt.; *p* = 0.034, d = 0.42). Furthermore, two of the low-functioning children, who did not perform any motor test before the program, were able to complete both reactive tests and transferring pennies test. No significant differences emerged for the remaining tests. Conclusions: A sport-based extra-curricular program improved reaction time and fine motor coordination in children with ASD. The complex reactive and transferring pennies tests were particularly effective in detecting changes, even in low-functioning children. These findings support the promotion of diverse physical activities to aid physical and cognitive development.

## 1. Introduction

Autism spectrum disorders (ASD) consist of various neurodevelopmental conditions distinguished by ongoing challenges in social communication and interaction across multiple settings, as well as by patterns of repetitive behaviors or limited interests or activities [1]. In Italy, the estimated prevalence of ASD among children aged 7 to 9 years is approximately 13.4 per 1000 (with a range from 11.3 to 16.0), and males show a higher prevalence than females [2]. Epidemiological data showed that the incidence has increased over the last five decades [3,4], and it is posing a significant challenge for global health [5].

The core features of ASD are impairments in social communication, according to the functioning level. Specifically, the Diagnostic and Statistical Manual of Mental Disorders, Fifth Edition (DSM-V) classifies ASD into three levels of severity. Level 1, the lowest severity classification, refers to high functioning and indicates that the individual requires support. Level 2 represents moderate severity level where individuals require substantial support. Level 3, classified as low functioning, is the highest severity level and indicates that the individual needs substantial support due to severe deficits in communication, behavior, social interaction, and daily living skills. Additionally, motor impairment is recognized as a common comorbid symptom, affecting up to 86.9% of children with an ASD diagnosis [6]. Despite this prevalence, motor deficits are not considering a diagnostic criterium for ASD and, therefore, are often under-recognized and under-treated [7]. Generally, children with ASD score a significantly lower performance on motor and gestural assessments that might decrease the opportunity for social interactions [8,9]. In particular, this population showed an impairment not only in coordination, postural stability, and gait patterns, but also in fundamental motor skills (FMS), which are essential for developing more advanced skills [10]. Research has shown that school-age children with ASD perform movement skills at approximately half the level of their typical developing (TD) peers, relative to their chronological age [11]. This motor delay, combined with reduced motivation or interest in physical activity and lack of social skills, often leads to physical inactivity [12]. However, physical activity has emerged as a promising non-pharmacological intervention for children with ASD, with evidence supporting its effectiveness in enhancing both motor skills and social functioning [12,13].

Target physical interventions can improve FMS and overall motor development [14,15], positively contributing to quality of life [16]. Nonetheless, much of the existing literature focuses on specific types of interventions and often lacks a comprehensive and inclusive approach [14,17].

To address these gaps, the present pilot study aimed to evaluate the effects of an inclusive extra-curricular sport-based training program on motor coordination and functional abilities in children with ASD. The intervention was designed not only to enhance motor performance but also to support the development of social and communication skills. By promoting inclusive physical activity involving TD peers and providing support through dedicated tutors, the program proposed in this study may offer children with ASD, particularly those with lower functioning, a structured opportunity to engage in physical activity, develop motor competencies, and improve social interaction. The approach in this pilot study may represent a valuable avenue for supporting both motor and communicative development in children with ASD. Based on these premises, it was hypothesized that children with ASD would show significant improvements in motor coordination, consistent with the current literature indicating that exercise interventions have positive effects on motor skills [18,19].

## 2. Materials and Methods

This study was approved by the Research Ethics Board of the University of Parma (Parma, Italy, reference number: 75674-6 March 2024) and conducted in accordance with the Declaration of Helsinki. Written informed consent was obtained from the legal representatives for all participants.

### 2.1. Participants

**Figure 1:**
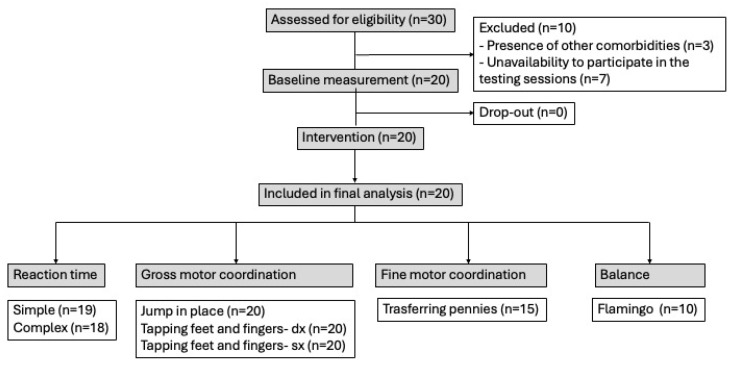
Flow chart of the experimental design of the study.

The recruitment of participants was conducted by the All Inclusive Sport association managed by the Volunteer Service Center (CSV) in Reggio Emilia in Italy. Among the 30 children with ASD enrolled in their program, only 20 were considered eligible for the participation. Their parents were invited in a meeting to receive all the information about the study design, the experimental procedures, and the objectives of the research project. The remaining 10 children were excluded for the following reasons: (a) presence of other comorbidities; (b) unavailability to participate in the testing sessions (Figure 1). After providing their acceptance for participation in the study, the parents signed informed consent. The final sample was composed of 15 males and 5 females with an average age of 8.7 ± 1.6 years.

### 2.2. Experimental Procedures

A one-group, pre-test post-test study design was conducted to investigate the effect of a sport-based training program in children with ASD. Pre- and post-intervention measurements were conducted before and after the 6-month intervention period. The evaluators were the same at pre- and post-testing sessions, and they were not blinded to the study aims. The participants attended a familiarization session one week prior to the beginning of the experimental procedures to receive the instructions of all the testing procedures and to practice the execution of the tests. Before each evaluation, the evaluators made sure that the children understood the task. To ensure the task comprehension at both baseline and post-intervention stages, trained staff provided verbal instructions and demonstrations. The children’s understanding was confirmed through practice trials before each test session. A testing battery was designed to evaluate reaction time and different domains of gross and fine motor coordination. The order of the tests consisted of the execution of two reactive tests, jumping in place (same side synchronized), tapping feet and fingers (same side synchronized), and transferring pennies, using the Bruininks–Oseretsky Test of Motor Proficiency, Second Edition, Short Form (BOT-2-SF) [20], and the Flamingo test [21]. All measurements were conducted in an indoor sport facility.

#### 2.2.1. Reactive Tests

**Figure 2:**
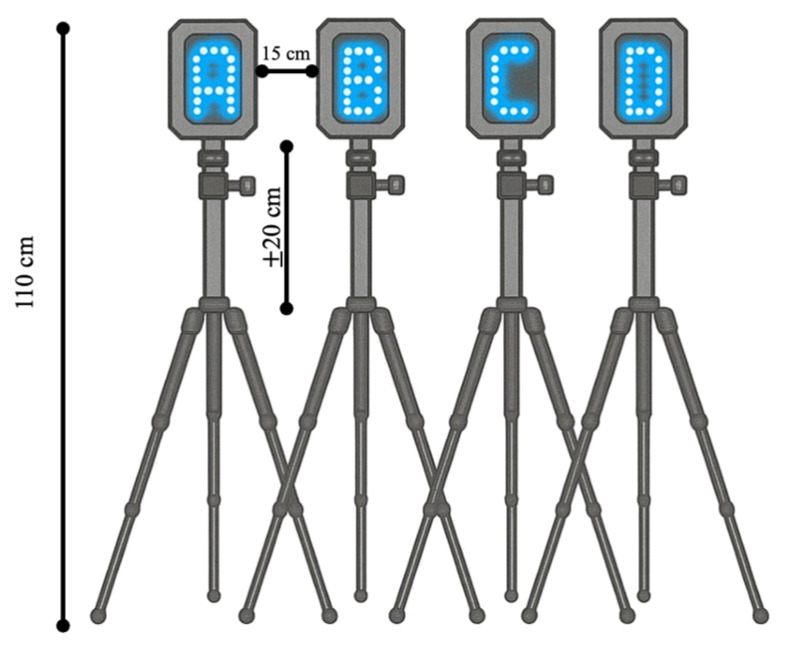
Schematic illustration of WittySEM configuration for the reactive tests.

Two reactive tests were conducted using a lighting system (WittySEM, Microgate Srl, Bolzano, Italy), typically used to investigate reaction time and speed processing in sport settings [22]. This method effectively assesses reaction time in children with mild intellectual disabilities (ID), providing precise and reliable data for analyzing the effectiveness of the plyometric training program [23,24]. Additionally, it evaluates reaction time in adolescents with special needs following a psychomotricity program [25]. The test setting consisted of 4 lighting devices, composed of a 5 × 7 LEDs matrix wirelessly connected to a chronometer. To determine the height of each device, we estimated the average height of children in the primary school age group, which was 129.6 cm. Based on this estimation, we set a standard height of 110 cm, with a standard deviation of ±20 cm. The 4 devices were positioned 15 cm apart from each other (Figure 2).

**Figure 3:**
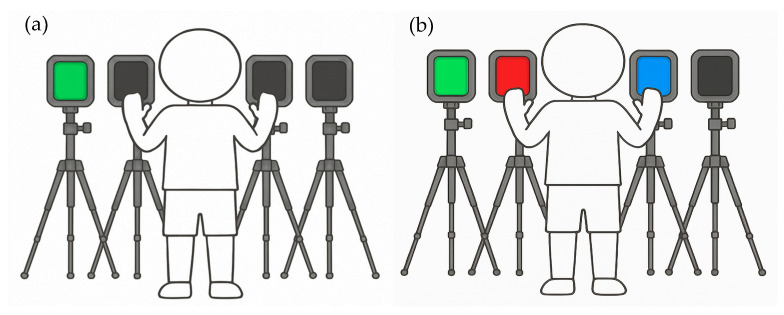
Schematic illustration of the simple reactive test (**a**) and the complex reactive test (**b**).

The reaction time was assessed with a simple and a complex reactive test [26]. In the simple reactive test, participants were required to identify the single lighted device (i.e., green color) among the four devices (Figure 3a). In the complex reactive test, participants were instructed to discriminate the targeted stimulus (i.e., green color) among other confounding visual stimuli (i.e., red and/or blue colors) (Figure 3b). Both tests involved the administration of 11 consecutive stimuli. The first stimulus served as the test onset and the total time was calculated for the 10 following stimuli. Participants were required to get one hand closer to the lighted device to turn it off. They were allowed to use both hands. Two trials were performed for each test, with a 2 min resting time in between, and the best trial was used for the statistical analysis.

#### 2.2.2. Jumping in Place and Tapping Feet and Fingers

Two tests of the BOT-2-SF subscale [20] were used to evaluate the gross motor coordination. For the jumping in place (same side synchronized), participants were asked to perform a maximum of five jumps in place. The number of correct executions performed consecutively was considered the performance outcome of the test. Two trials were performed, with a 2 min resting time in between, and the best trial was used for the statistical analysis.

For the tapping feet and fingers (same side synchronized), participants were asked to perform a maximum of ten synchronized taps, both with their feet on the ground and with their index finger on a table, one time with the right side and one time with the left side. The number of correct synchronized taps performed consecutively was considered the performance outcome of the test. Two trials were performed for each test and side, with a 2 min resting time in between, and the best trial for each condition was considered for the statistical analysis.

#### 2.2.3. Transferring Pennies

The transferring pennies [20] test was used to measure fine motor coordination. The test involved 20 pennies and one box. The participants were instructed to take a penny with their dominant hand, transfer it to their non-dominant hand, and then place the penny in the box. After placing a penny in the box, the participant could continue with the next penny. The number of pennies successfully transferred and inserted in the box in 30 s was considered the performance outcome of the test. Two trials were performed, with a 2 min resting time in between, and the best trial (i.e., the highest number of pennies) was used for the statistical analysis.

#### 2.2.4. Flamingo Test

Balance was assessed using the Flamingo test [21]. Participants were required to maintain a single-leg stance for one minute with their dominant leg. The final score consisted of the number of foot contacts with the ground used to regain on maintain balance. Only one trial was allowed for each participant.

### 2.3. Intervention

A multidisciplinary team, consisting of neuropsychiatrists from the Local Health Unit of Reggio Emilia (AUSL, Reggio Emilia, Italy) and families, selected an extra-curricular activity for the children based on their interests and needs. Each child with ASD participated in the program alongside TD peers, and a trained tutor was assigned to each child with ASD to follow and help them during the training. Table 1 summarizes the various sports chosen and the number of minutes of each activity per week. As illustrated, the activities are diverse and vary among children, depending on the specific organization of each sports club. Total weekly training volume was recorded for each participant by the tutors using attendance logs to track participants’ presence. The total week volume had a mean of 110 min per week (range: 50–270 min).

### 2.4. Statistical Analysis

Statistical analysis was conducted using the Statistical Package for the Social Sciences software (version 27, IBM Corp., Armonk, NY, USA). The significance level was set at *p* < 0.05. All results are presented as mean ± standard deviation (SD), median, and interquartile range (IQR). Prior to the analysis, the Shapiro–Wilk test was applied to ascertain the normality of data distribution. A non-parametric test was used to analyze all data due to the non-normal distribution of data and the sample size. The Wilcoxon signed-rank test was applied to ascertain differences between pre- and post-intervention measurements. The effect size was measured using Cohen’s value for parametric testing and interpreted as trivial (<0.19), small (0.20–0.59), moderate (0.60–1.19), large (1.20–1.99), very large (2.0–4.0), and extremely large (>4.0). The effect size for non-parametric testing (r) was reported and interpreted as small (0.1), moderate (0.3), and large (0.5).

## 3. Results

**Figure 4:**
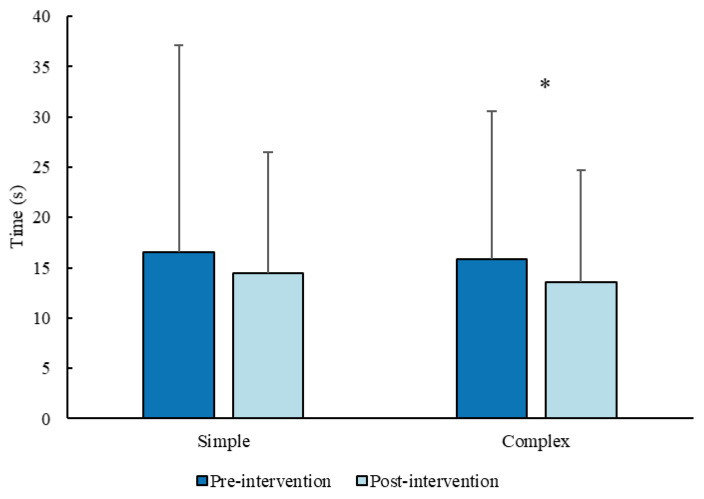
Total reactive time (mean ± SD) from the 10 stimuli for the simple (*n* = 19) and complex reactive test before and after the intervention (sample *n* = 18). * *p* < 0.05.

For the reactive tests, a significant difference (*p* = 0.016, r = 0.55) between the pre- (mean ± SD; median; interquartile range: 15.8 ± 14.8 s; 9.8 s; 8.1–17.5 s) and post- (13.6 ± 11.1 s; 10.4 s; 7.4–15 s) intervention measurements emerged only for the complex reactive test (Figure 4). No differences (*p* = 0.526) emerged for the simple test between the pre- (16.5 ± 20.6 s; 7.7 s; 7.1–17.8 s) and post- (14.5 ± 12 s; 9.4 s; 6.7–17.4 s) intervention measurements.

**Figure 5:**
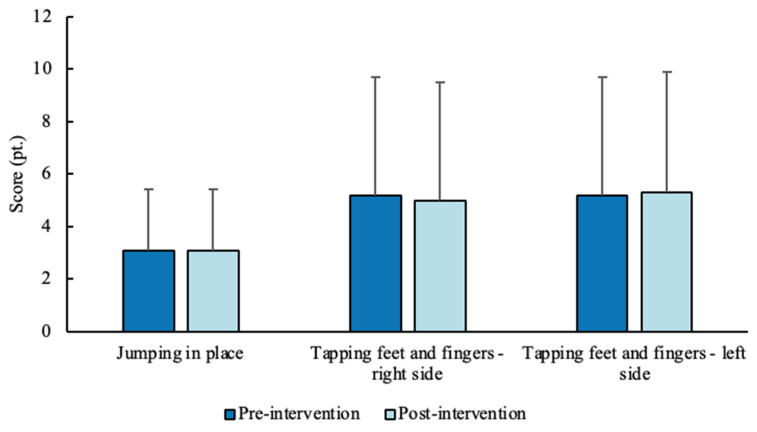
Score (mean ± SD) for the three gross motor coordination tests before and after the intervention (sample *n* = 20).

For gross motor coordination, no significant differences (jumping in place: *p* = 0.713; right-side tapping feet and fingers: *p* = 0.905; left-side tapping feet and fingers: *p* = 0.953) were found between the pre- (jumping in place: 3.1 ± 2.3 pt.; 4.5 pt.; 0–5 pt.; right-side tapping feet and fingers: 5.2 ± 4.5 pt.; 5 pt.; 0–10 pt.; left-side tapping feet and fingers: 5.2 ± 4.5 pt.; 4 pt.; 0–10 pt.) and post- (jumping in place: 3.1 ± 2.3 pt.; 4.5 pt.; 0–5 pt.; right-side tapping feet and fingers: 5 ± 4.5 pt.; 5 pt.; 0–10 pt.; left-side tapping feet and fingers: 5.3 ± 4.6 pt.; 4.5 pt.; 0–10 pt.) intervention measurements (Figure 5).

**Figure 6:**
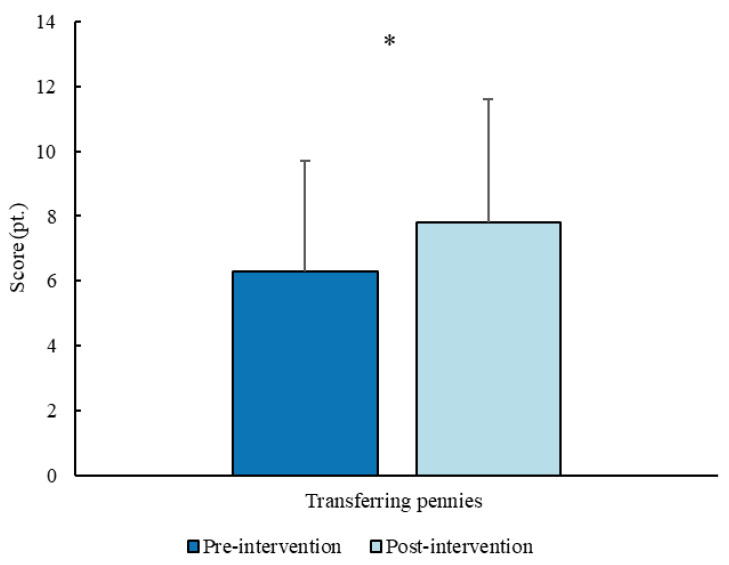
Score (mean ± SD) for the fine motor coordination test before and after the intervention. * *p* < 0.05.

For fine motor coordination, transferring pennies test showed a significant increase (*p* = 0.034, d = 0.42) from the pre- (6.3 ± 3.4 pt.; 6 pt.; 3–7 pt.) to the post- (7.8 ± 3.8 pt.; 8 pt.; 5.5–10 pt.) intervention measurements (Figure 6).

**Figure 7:**
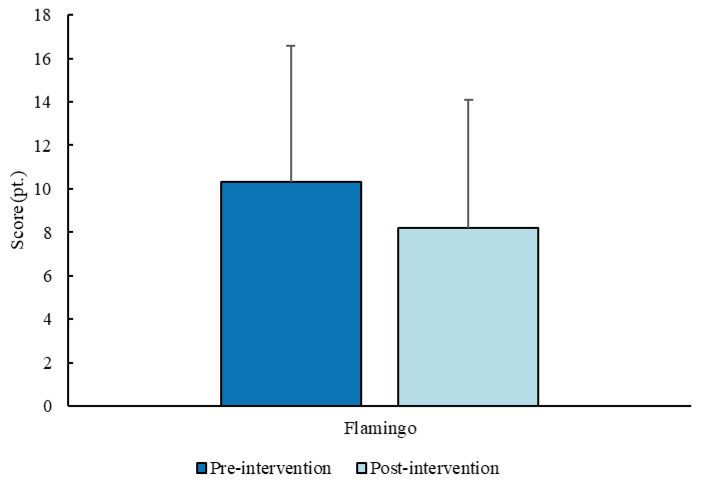
Score (mean ± SD) for the balance test before and after the intervention (sample *n* = 10).

For the Flamingo test, no significant differences (*p* = 0.066) were found between the pre- (10.3 ± 6.3 pt.; 11 pt.; 6.5–13 pt.) and post- (8.2 ± 5.9 pt.; 7 pt.; 3.7–11.2 pt.) intervention measurements (Figure 7).

## 4. Discussion

The main purpose of this pilot study was to determine the effect of a sport-based training program on motor coordination and functioning in children with ASD. The main findings indicated a significant enhancement observed in complex reactive test and fine motor coordination. Notably, two children who were unable to complete the motor assessments at baseline successfully performed both the reactive tasks and the transferring pennies test following the intervention. No significant changes were detected in the remaining tests.

The sample of the current pilot study confirms the common trend of the prevalence of males with a diagnosis of ASD compared with females. The gender difference that characterizes ASD is due to biological and diagnostic factors. Regarding biological factors, evidence from animal models indicates that female sex hormones contribute to this process [27]. Certain sex hormones appear to exert neuroprotective effects and may mitigate specific abnormalities within the nervous system. Such mechanisms may partly account for the divergent manifestations of ASD in females compared to males, frequently contributing to underdiagnosis in the female population [28].

The evaluation of a simple and complex reactive task showed different results. The simple reactive test demonstrated a tendency of improvement and a reduction in standard deviation at the post-intervention measurement, highlighting a lower variability among the sample. It can be speculated that the simpler nature of the task was not sufficiently sensitive to detect small meaningful changes before and after an intervention program. Moreover, the distinct characteristics of the different training programs adopted by each participant might have further influenced the potential effect of an intervention program on a simple task. Conversely, a significant difference emerged for the complex reactive test, underlining the effective impact of the participation in different sport-based training programs. The reduction in reaction time aligns with Gogean Groza’s [24] findings, which showed a significant decrease in reaction time after a plyometric training program in children with ID. Similar evidence demonstrated that cognitive–motor dual task training improves motor skills and response speed in a cognitive test in children with ASD compared to children with TD [29].

The observed improvements in reaction time may reflect enhanced cognitive–motor integration rather than isolated gains in motor execution speed. Reaction time performance depends on the efficient coupling of perceptual processing, decision making, and motor planning, which are core components of cognitive–motor integration [30]. From this perspective, the faster responses observed in the present pilot study suggest a more efficient coordination between cognitive and motor processes. This interpretation is consistent with evidence showing that tasks requiring simultaneous cognitive engagement and motor or postural control promote integrative adaptations within shared neural networks, leading to improved response efficiency [31,32].

The participation in sport-based training program induced effects in neuromuscular response and coordination. Specifically, we found only a significant improvement in fine motor coordination, while the results for gross motor coordination did not show a significant difference before and after the sport-based training program. In particular, the performance of jump in place remained stable across the two measurement points. Meanwhile, tapping feet and finger tests showed a slight decline on the right side and a small improvement on the left side. These results might be attributable both to the heterogeneity of the sport programs and the substantial interindividual variability typically observed in children with ASD. It is plausible that the program preferentially enhanced fine motor coordination, whereas improvements in gross motor coordination may require a more structured and specifically target intervention to produce significant enhancement.

A recent study showed that motor skills performance was influenced by several factors, including postural sway area, diagnosis of ASD, and chronological age [33]. Moreover, postural stability has been considered a prerequisite for the development of gross motor skill patterns [33], and for compensating motor deficits [34], suggesting that the underlying balance score may partially explain the observed results. The balance test did not show a significant enhancement. In fact, children with ASD generally present an underdevelopment of postural control compared to TD peers [35]. One explanation relies on the underconnectivity theory, which ties into the underdevelopment of integrative circuitry and the emergence of cognitive, perceptual, and motor skills in people with ASD [36]. Moreover, it has been suggested that worsened balance skills could be linked to anxiety [37]. However, several studies have shown that physical interventions explicitly targeting postural control can improve balance, unlike our program, which did not focus exclusively on postural stability [34,38,39,40]. This difference in targeted approach may, therefore, account for the divergence observed in our results, which likely stems from the distinct type of intervention employed.

It is worth mentioning that some participants were excluded from the analysis because they failed to complete some tests during the pre-intervention measurements. However, they were able to fully execute the reactive tests and the transferring pennies task after the intervention; these results provide preliminary evidence for qualitative functional gain which differ from the quantitative improvement observed in children who completed the assessments at both pre- and post-intervention testing session. Although the post-intervention measurements could not be compared with the value at baseline, it can be speculated that the capability to complete a required task could be explained as a real functional improvement in the children. Moreover, the compliance observed in the reactive tests suggests that integrating new technologies and testing procedures based on lighting system could represent a new approach to assess motor–cognitive functioning in this population. Additionally, recent studies have shown that technology, such as virtual reality or advanced robotics, can improve joint attention [41] and motor domains [42] in children with ASD, consistent with the improvement we observed during the cognitive assessments involving the reactive test.

The observed changes should be interpreted with caution as they may be partly attributed to factors unrelated to the intervention. Specifically, maturation effects may have contributed to improvements over time, as participants could naturally change or develop independently of the experimental manipulation. It is also essential to consider the effects of repeated testing, as increased familiarity with the assessment tools may have influenced performance in follow-up evaluation. Overall, these factors represent plausible alternative explanations that should be considered when interpreting the results. Furthermore, it is important to emphasize that the findings should be viewed as performance-based indicator rather than validated clinical measures. While the results offer preliminary insight into functional changes and trends, they do not constitute formal clinical assessments. This distinction highlights the need for caution in interpreting the practical implication of the study.

It is essential to emphasize that in this pilot project, children with ASD participated in sports activities alongside TD peers, with the aim to promote socialization and inclusion in sports clubs. This pilot study aligns with the existing literature, indicating that the involvement of TD students in the inclusive physical activity program alongside peers with ASD did not negatively impact either group [43]. Furthermore, a notable improvement was observed in the fundamental motor skills of the students who participated in the inclusive physical activity program [44]. In our project, we focused on promoting ecological sports participation in natural and inclusive environments. Our goal was to enhance engagement, motivation, and functional abilities. This approach differs from traditional structured motor learning interventions, which concentrate on specific skills acquisition through guided practice. While those methods may improve particular motor skills, they often do not lead to broader functional outcomes. Therefore, implementing inclusive physical activity with curricular and extra-curricular activities could be beneficial for socialization and inclusion of children with disabilities. However, to fully understand the impact of such interventions, future research should also investigate the behavioral and social outcomes associated with inclusive physical activity.

This pilot study has some limitations that should be highlighted. First, the relatively small sample size may have constrained the results, and the absence of a control group was due to logistical challenges (particularly related to participant recruitment). Additionally, the significant variability in activities and the amount of exposure time to the intervention further limited the interpretation and generalizability of the findings. The observed effects should not be attributed to any specific sport modality; rather, they appear to be associated with participation in inclusive, sport-based activities. Such participation may promote psychomotor, cognitive, and social benefits, regardless of the specific sport practiced. Second, the absence of direct observation of sports activities by the researchers, in addition to the tutor’s involvement, limited the assessment of adherence to children’s participation and behaviors. Third, the variability in the participants’ levels of functioning may have also influenced the results, introducing a level of heterogeneity that was not fully captured in the analysis. In addition, multiple outcomes were reported with basic data visualization and without formal adjustment due to the exploratory nature of the study. However, this approach may have increased the risk of type I error; thus, the results should be interpreted with caution. Lastly, the reactive tests adopted in this study have not been validated specifically for children with ASD, as there are currently no validated tests in the literature. This could affect the accuracy and interpretability of the findings.

## 5. Conclusions

This pilot study provides preliminary evidence that a sport-based training program was associated with improvements in reaction time and fine motor coordination in children with ASD. It highlights the complexity of motor outcomes within this population. The significant gains observed in a complex reactive task suggest that cognitively demanding and motivating activities may play a crucial role in enhancing neuromuscular responsiveness and coordination.

However, the limited improvements in gross motor coordination and balance emphasize the influence of developmental, neurological, and diagnostic factors that may hinder progress in these areas. Despite the lack of significant changes, the ability to finalize the execution of a test during the post-intervention measurements in some participants provides preliminary evidence of meaningful functional progress, with several children showing indications of new task competencies following the intervention.

These preliminary results support the importance of integrating children with ASD into inclusive sport-based training programs, which not only foster motor skill development but also promote socialization and inclusion alongside TD peers.

Nonetheless, the outcomes should be interpreted with caution due to the study’s limitations, including a small sample size, variability in participant functioning, and the absence of validated tests specific for ASD. Future research should aim to replicate these findings with larger samples. Additionally, the integration of motor outcomes with neuropsychological and socio-adaptive outcomes will provide a more comprehensive understanding of how adapted and inclusive physical activity can support the development of children with ASD.

Overall, this pilot study adds evidence to the growing body of literature supporting inclusive physical activity as a promising approach.

## Figures and Tables

**Table 1:** Overview of the sports practiced and weekly hours of activity.

Sport-Based Training Program	*n*	Min/Week
Exercise	2	60
Baskin	2	90
Athletics	2	90
Judo	4	100
Ice-skating	2	270
Swimming	4	50
Football	1	180
Climbing	1	60
Dance	1	60
Artistic gymnastics	1	90

## Data Availability

The data presented in this study are available on request from the corresponding author. The data are not publicly available due to privacy restrictions.

## Abbreviations

The following abbreviations are used in this manuscript:

ASD	Autism spectrum disorders
ID	Intellectual disabilities
TD	Typical developing
FMS	Fundamental motor skills
